# Identification and Phylogenetic Analysis of *Flavobacterium* spp. Associated with Aquaculture Fish Diseased from Brazil

**DOI:** 10.3390/pathogens14030219

**Published:** 2025-02-22

**Authors:** Peter Charrie Janampa-Sarmiento, Henrique Lopes Costa, Júlio César Câmara Rosa, Guilherme Campos Tavares, Henrique César Pereira Figueiredo

**Affiliations:** Laboratory of Aquatic Animal Diseases-Aquavet, Department of Preventive Veterinary Medicine, School of Veterinary Medicine, Federal University of Minas Gerais—UFMG, Belo Horizonte 31270-901, MG, Brazil; peterjs_0126@hotmail.com (P.C.J.-S.); henriquelopes.costa17@gmail.com (H.L.C.); jcbhrama@gmail.com (J.C.C.R.); gcamposvet@hotmail.com (G.C.T.)

**Keywords:** clinical bacteriology, columnaris disease, freshwater fish, multiplex PCR, proteomics

## Abstract

Due to the recent taxonomic reclassification of the species *Flavobacterium columnare* into four new species—*Flavobacterium columnare*, *Flavobacterium davisii*, *Flavobacterium covae*, and *Flavobacterium oreochromis*—it is necessary to re-evaluate isolates of previous outbreaks to better understand the epidemiology related to this bacterial group. Therefore, the aim of this study was to determine the taxonomic profile of Brazilian isolates of *Flavobacterium* spp. associated with columnaris disease using available diagnostic methods. Fifty isolates from different outbreaks (17 clinical cases) occurring in five different Brazilian states previously identified as *F. columnare* were selected and identified by multiplex PCR and MALDI-ToF methods. In addition, at least one isolate from each clinical case was analyzed by *16S rRNA* gene sequencing. After inclusion of the MSPs (main spectra profiles), the isolates were identifiable, and when compared with the multiplex PCR results, they showed almost perfect agreement (94.2% Kappa = 0.85). Only *F. davisii*, *F. covae*, and *F. oreochromis* were found among the Brazilian isolates, with these species causing disease in neotropical fish hosts not previously reported (e.g., Siluriformes, Serrasalmidae, and Bryconidae), while *F. columnare* was not detected. This study provides evidence of *Flavobacterium* species associated with columnaris disease circulating in various aquaculture facilities across different regions of Brazil. This information is crucial for developing control programs and advancing epidemiologic studies on columnaris disease in Brazilian aquaculture.

## 1. Introduction

The *Flavobacterium* genus includes 330 species validly published under the International Code of Nomenclature of Prokaryotes [1]. Several of these *Flavobacterium* species are host-associated in fish [2], including *Flavobacterium columnare*, which was appointed as the causative agent of columnaris disease in cold, temperate, and tropical fish from both wild and aquaculture freshwater environments [3].

Columnaris disease was described first in 1922 and manifests as lesions on the body surface and gills, appearing as dirty-white or yellowish areas on diseased fish. As the disease progresses, it can lead to high mortality rates [3,4]. This disease has a significant economic impact on various aquaculture production systems for farmed fish species worldwide [3,5,6,7].

Since the first successful isolation of the bacterial agent of columnaris disease in 1944 [8], its definitive taxonomy was believed to have been established in 1996 with the designation of *Flavobacterium columnare* [9]. However, a recent study has demonstrated that *Flavobacterium columnare* can be distinguished into four species: *Flavobacterium columnare*, *Flavobacterium davisii*, *Flavobacterium covae*, and *Flavobacterium oreochromis* [10]. Species identification of these new species of *Flavobacterium* bacteria associated with columnaris disease was originally conducted using phylogenetic and phylogenomic analyses [10]. Additionally, multiplex PCR targeting *Flavobacterium columnare* genotypes and MALDI-TOF MS have been proposed as methods for distinguishing *Flavobacterium* species associated with columnaris disease [10,11], and they have been applied in recent reports [12,13,14,15,16,17,18].

Columnaris disease was suspected to occur in Brazilian aquaculture as early as the late 20th century [19], but it was only isolated and characterized around 2005 from an outbreak in Nile tilapia (*Oreochromis niloticus*) [20,21,22]. Since then, reports of columnaris disease associated with *F. columnare* isolation and DNA sequencing and phylogenetic identification in other fish species have been registered in the country [5,13]. Nowadays, columnaris disease is an important disease for Brazilian freshwater aquaculture, affecting *O. niloticus* and some neotropical fish species, especially during the fingerling phase or after fish are exposed to environmental stress (such as water temperature reduction) or management stress (such as fish transport) [13,17,23].

Pathogenicity capabilities of *F. columnare* Brazilian isolates for *O. niloticus* and neotropical fish have already been proven [13,17,21,24,25]. However, with the new division of *F. columnare* into four taxonomic groups, a new epidemiologic approach for columnaris disease can be applied according to the specific *Flavobacterium* species involved. For instance, some authors have suggested that the incidence of *F. oreochromis* and *F. covae* is more closely associated with Nile tilapia and channel catfish, respectively [12,26].

Given the recent classification of *F. columnare* into four species associated with columnaris disease, it is essential to update the classification of isolates previously identified as *F. columnare*. This study aimed to determine the taxonomic profiles of Brazilian columnaris-causing bacteria through phylogenetic analyses and MALDI-ToF MS.

## 2. Materials and Methods

### 2.1. Bacterial Isolates

Isolates previously identified as *F. columnare* using species-specific PCR [27] were selected from the culture collection of the Laboratory of Aquatic Animal Diseases (AQUAVET) (*n* = 46) and the Laboratory of Applied Microbiology of Aquatic Organisms (LAMAO) (*n* = 4), as well as the reference strains ATCC23463 and ATCC49512. The selected isolates were isolated between 2004 and 2022 from columnaris disease outbreaks in five different states (Amazonas, Minas Gerais, Mato Grosso, Rondônia, and São Paulo) and from six different host species (*Colossoma macropomum Lophiosilurus alexandri*, *Oreochromis niloticus*, and *Brycon amazonicus* and the hybrids *Colossoma macropomum × Piaractus brachypomus* and *Leiarius marmoratus × Pseudoplatystoma fasciatum*) (Table 1). The isolates and reference strains that were preserved in Hsu–Shotts broth (MHS) with 15% glycerol and maintained at −70 °C were thawed, streaked, and incubated in Hsu–Shotts agar (MHSA) at 25 °C for 5 days.

### 2.2. Molecular Biology Analysis

#### 2.2.1. DNA Extraction

Colonies of all isolates and strains of *Flavobacterium* spp. were collected from agar plates after incubation using sterile 10 uL plastic loops and subjected to DNA extraction using the Maxwell^®^ 16 Tissue DNA Kit on the Maxwell 16 Research Instrument (Promega, Madison, WI, USA), following the manufacturer’s instructions. The total DNA underwent quality and quantity verification with the aid of the NanoDrop spectrophotometer (Thermo Fisher Scientific, Waltham, MA, USA); those samples with purity values of 1.8 ± 0.5 were stored at −70 °C until use.

#### 2.2.2. *16S rRNA* Gene Sequencing

To determine the species of *Flavobacterium* involved in different columnaris disease outbreaks, the DNA of 29 isolates from those extracted was selected, along with that of two reference strains, ATCC23463 and ATCC49512. In order to represent the different affected farms, at least one isolate from each clinical case was selected randomly (Table 1). The *16S rRNA* gene was sequenced following the polymerase chain reaction (PCR) using the universal primers B37 (5′-TACGGYTACCTTGTTACGA-3′) and C70 (5′-AGAGTTGATYMTGGC-3′), exactly as reported by Fox et al. [30].

The obtained amplicons were purified using Agencourt AMPure XP (Beckman Coulter, Pasadena, CA, USA), as recommended by the manufacturer, and sequenced with the BigDye™ Terminator v.3.1 Cycle sequencing kit (Applied Biosystems, UK) and an ABI 3500 genetic analyzer (Life Technologies, USA). Subsequently, quality control, sequence trimming, and contig generation were performed using BioEdit, version 7.2 [31], as follows: visual inspection of chromatograms, followed by the removal of nucleotide bases with suboptimal or noisy peaks in the ABI trace file for each forward and reverse read [32], and, finally, contig assembly using the CAP application (minimum base overlap = 20, minimum percent match = 85). All assembled sequences were evaluated using NCBI BLAST version 2.15.0 (https://blast.ncbi.nlm.nih.gov/Blast.cgi, accessed on 1 February 2025) on the nt/nr database with the default parameters of the *megablast* algorithm. After the BLAST procedure, identity (greater than 97%) and coverage (greater than 99%) were determined based on the lowest significant E-values.

#### 2.2.3. Multiplex PCR and Amplicon Sequencing

All extracted DNA was also subjected to multiplex PCR to determine the species, using the only the available protocol for species differentiation among columnaris-causing bacteria, as described by LaFrentz et al. [11], with some modifications. Briefly, multiplex PCR assays were performed with the HotStart GoTaq DNA polymerase kit (Promega, Madison, WI, USA), with 50 ng of bacterial DNA per reaction. To carry out the multiplex PCR, there was an initial denaturation step at 95 °C for 5 min, followed by 20 cycles of denaturation at 95 °C for 30 s, an annealing step at 54 °C for 20 s, and an extension phase at 72 °C for 60 s. The final extension step was performed at 72 °C for 5 min. Thermal cycling was performed in a 96-well Veriti^®^ thermal cycler (Life Technologies). The resulting amplicons were assessed for size using a QIAxcell Advanced and a QIAxcell DNA Screening Kit (Qiagen, Valencia, CA, USA). The primer sequences for multiplex PCR used in this study, as well as the amplicon sizes, are displayed in Supplementary Table S2.

For multiplex PCR sequencing, two random amplicons of clinical samples were chosen for only three available product sizes (320, 659, and 894 bp). These six samples were then subjected to sequencing with the primers of interest for further evaluation, as described in Section 2.2.2.

#### 2.2.4. Phylogenetic Analysis

Phylogenetic analysis was performed on the sequences of the 16S rRNA gene obtained from the Brazilian isolates (*n* = 29) and other *Flavobacterium* spp. obtained from the NCBI database (*n* = 26). For this purpose, sequence alignment was performed on MAFFT v7.471 (settings: —adjustdirection—auto—reorder) [33], followed by phylogenetic tree building in MEGA7 [34] by fitting the General Time-Reversible model [GTR + G + I] based on the maximum likelihood (ML) method. All positions containing gaps and missing data were eliminated, leaving a total of 1255 positions in the final dataset submitted to MEGA7 (bootstrap = 1000, Discrete Gamma categories = 5, ML Heuristic Method = NNI, Initial Tree for ML = default NJ/BioNJ, Branch Swap Filter = none). The final phylogenetic tree was edited in FigTree v.3.4.4 software [35] and rooted using *Capnocytophaga ochracea* (strain ATCC 27872T; Accession Number: U41350) as an external group.

### 2.3. Mass Spectrometry by MALDI-ToF Analysis

#### 2.3.1. Custom Main Spectra Profile (MSP) Creation

In order to identify the four species of *Flavobacterium* by the MALDI-ToF MS method, using the Bruker MALDI Biotyper database (v7), the first step was to insert a main spectra profile (MSP) of each one. For this, after detection of the species by multiplex PCR and confirmation by Sanger sequencing (Supplementary Table S1), representative isolates were determined (FC05—*F. covae*, FC14—*F. davisii*, FC27—*F. oreochormis*, and ATCC49512—*F. columnare*) for inclusion in the device database as standard MSPs. The strains that had been previously cultivated in MHSA (as mentioned in Section 2.1) were collected to perform protein extraction, which was performed as described in the previous works of the AQUAVET research team [36,37].

For spectra acquisition and evaluation, 1 μL of the supernatant obtained from protein extraction was added to 8 different spots on the MSP 96 target polished steel plate. After drying, 1 μL of the α-cyano-4-hydroxycinnamic acid (HCCA) matrix was added. Triplicate readings were performed at each point to obtain 24 spectra, via the FlexControl MicroFlex LT software (version 3.4, Bruker Daltonics), which were then analyzed using the FlexAnalysis software (Bruker Daltonics). The 3 worst-quality spectra were discarded, while the selected spectra were forwarded to the MALDI Biotyper 3 software (Bruker Daltonics) to create the MSPs. Those MSPs that, when evaluated by the software quality control, presented a minimum score of 2.700 and a peak frequency of 75% were considered approved. The protocols were performed according to the manufacturer’s recommendations.

#### 2.3.2. Bacterial Identification by MALDI-ToF MS

The *Flavobacterium* isolates underwent the identification process after the creation and inclusion of the MSPs. For this, the 50 isolates already cultivated in MHSA had between 1 and 3 colony forming units (CFUs) collected with the aid of a sterile disposable wooden toothpick and placed in an identification spot on the MSP 96 target polished steel plate. Then, 1 µL of formic acid (70%) was added, and after the solution dried, 1 µL of HCCA matrix was applied and allowed to dry naturally. All spectra were acquired using the FlexControl MicroFlex LT software (Bruker Daltonics) with the same parameters described in Assis et al. [22]. The standard bacterial test (BTS) (*E. coli* DH5 alpha; Bruker Daltonics) was used to calibrate the mass detection. The scoring criteria for confirming the microorganisms were those recommended by the manufacturer, where scores ≥ 2000 indicate identification of the species, scores ≥1700 and <2000 indicate identification relative to the genus, while scores < 1700 are considered as an absence of reliable identification.

### 2.4. Statistical Analysis

Comparisons between the multiplex PCR and MALDI-TOF MS identification methods were performed using Jamovi software version 1.6 [38,39,40], with agreement rates determined by the Kappa coefficient (ρ < 0.05).

## 3. Results

### 3.1. Species Identification by 16S rRNA Gene Sequencing

**Figure 1:**
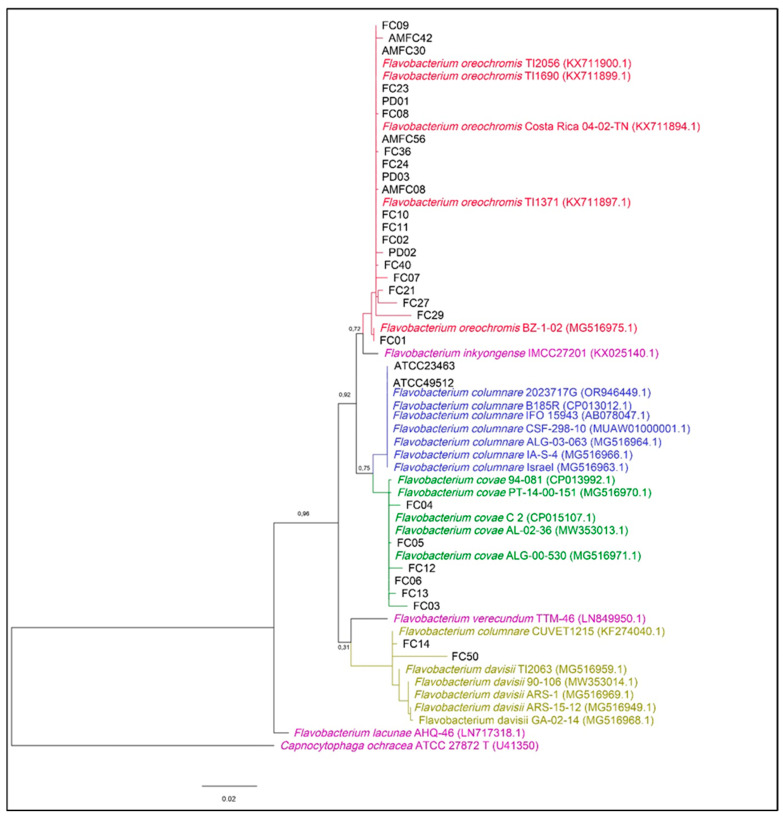
Phylogenetic tree based on *16S rRNA* gene sequences showing the phylogenetic positions of Brazilian isolates (in black) among the four *Flavobacterium* species reported by LaFrentz et al. [10] (*F. oreochromis* in red, *F. columnare* in blue, *F. covae* in green, and *F. davisii* in yellow) and other Flavobacteriaceae isolates (in purple). Relatedness was inferred using the maximum likelihood method based upon the General Time-Reversible model [GTR + G + I; 50] and rooted with *Capnocytophaga ochracea* ATCC 27872T. The percentage of trees in which the associated sequences clustered together in the bootstrap test (1000 replicates) is shown next to the main branches. The analysis involved 58 nucleotide sequences, all positions containing gaps and missing data were eliminated, and there was a total of 1255 positions in the final dataset.

The *16S rRNA* gene of 29 Brazilian isolates and 2 *F. columnare* ATCC references were successfully sequenced and identified as *Flavobacterium* species after the BLAST procedure (Table 1). Identity evaluation showed that *F. oreochromis* (13/17 clinical cases, 76.5%) and *F. covae* (3/17 clinical cases, 17.6%) were present in columnaris disease cases of Brazilian aquaculture fish. Additionally, there were two isolates (FC14 and FC50) that displayed high identity (> 98.9%) with strain CUVET1215, which was registered as *F. columnare* in the NCBI database. A further phylogenetic evaluation (Figure 1) showed that both these isolates, FC14 and FC50, and the strain CUVET1215 were all clustered inside the branch of *F. davisii* (2/17 clinical cases, 11.8%), as highlighted in a previous study [41]. Interestingly, although most clinical cases evaluated (*n* = 17) displayed isolates belonging to just one *Flavobacterium* species, there was one clinical case (Clinical Case 6) involving two species simultaneously (*F. davisii* and *F. covae*) in an outbreak in *O. niloticus* (Table 2).

Figure 1 displays a phylogenetic tree constructed with 29 Brazilian isolates, representing all 17 clinical cases of *Flavobacterium* spp. reported in this study. The tree also includes *16S rRNA* gene sequences from 20 *Flavobacterium* sp. fish strains used by LaFrentz et al. (2022) [3] and 5 additional Flavobacteriaceae strains available in the NCBI database: *F. columnare* (strain CUVET1215; Accession Number: KF274040.1), *Capnocytophaga ochracea* (strain ATCC 27872-T; Accession Number: U41350), *F. lacunae* (strain AHQ-46; Accession Number: LN717318.1), *F. inkyongense* (strain IMCC27201; Accession Number: KX025140.1), and *F. verecundum* (strain TTM-46; Accession Number: LN849950.1). Overall, all Brazilian isolates clustered within the phylogenetic branches of *F. oreochromis* (*n* = 21), *F. covae* (*n* = 6), and *F. davisii* (*n* = 2), with high bootstrap support (≥0.7).

### 3.2. Species Identification by Multiplex PCR and MALDI-TOF MS

Of the 52 isolates identified by multiplex PCR, 51 had their species determined by the technique. The results showed that the species *F. oreochromis*, *F. covae*, *F. davisii*, and *F. columnare* were identified in 76.92% (*n* = 40), 11.54% (*n* = 6), 5.77% (*n* = 3), and 3.85% (*n* = 2) of the isolates, respectively. Only isolate PD02, 1.92%, did not present any detectable amplicon, its identification not being allowed by the method (Table 1). Additionally, multiplex PCR amplicon identities of six selected isolates were confirmed by BLAST analysis (Supplementary Table S1). As expected, two isolates each of *F. davisii, F. covae*, and *F. oreochromis* were identified, in agreement with the amplicon size and Flavobacterium species described by LaFrentz et al. [10,11].

In order to evaluate the applicability of the MALDI Biotyper in performing the identification of *Flavobacterium* isolates, an MSP was created for each of the four species analyzed in this study using the isolates FC05—*F. covae*, FC14—*F. davisii*, FC27—*F. oreochormis*, and the reference strain ATCC49512—*F. columnare*. The MSPs presented a range of m/z peaks from 3086.50 to 13922.38, X to Y, with reproducibility between 76.2% and 100%, and were considered suitable for identification of the strains.

After the inclusion of MSPs, 98.08% (*n* = 51) of the isolates were identified at the species level, with mean, maximum, and minimum scores of 2.226, 2.022, and 2.601, respectively (Table 1). Isolate FC32 was not identified by MALDI at the species level; only the genus was reliably identified (score = 1.917) (Table 1). The proportions in relative frequencies were preserved in relation to the results obtained by multiplex PCR. The identification methods by multiplex PCR and MALDI-TOF MS were compared using the Kappa agreement method; it was found that the two methods tested obtained 94.2% agreement (Kappa = 0.852 and ρ < 0.001); this value indicates an almost perfect agreement.

### 3.3. Epidemiological Dispersion of Flavobacterium spp.

The observed frequencies of identified *Flavobacterium* species by origin and host are shown in Table 2. *F. oreochromis* was the only species present in all five Brazilian states considered in this study. In addition, this species was mainly isolated from diseased *O. niloticus* (6/17 clinical cases) and the neotropical fish *C. macropomum* (5/17 clinical cases). Other neotropical fish species, such as *B. amazonicus* (1/17 clinical cases) and *Colossoma macropomum × Piaractus brachypomus* (1/17 clinical cases), were also associated with fish disease by *F. oreochromis*. A similar host diversity infection was observed with *F. covae*, which was isolated from *O. niloticus* and two siluriform species (*L. alexandri* and *L. marmoratus* × *P. fasciatum*), but with a lower observed frequency (all with 1/17 clinical cases) and restricted to only one Brazilian state. Finally, *F. davisii* was only isolated from diseased *O. niloticus* (2/17 clinical cases) and from one Brazilian state.

## 4. Discussion

The etiology of columnaris disease in fish was recently associated with four different *Flavobacterium* species [3]. This study showed that Brazilian *Flavobacterium* isolates associated with columnaris diseases in aquacultured fish belong to at least three new species (*F. covae*, *F. davisii*, and *F. oreochromis*) described by LaFrentz et al. [3]. Interestingly, none of the Brazilian isolates reported in this study were identified as *F. columnare* by biomolecular diagnostic tools (*16S rRNA* gene phylogeny and multiplex PCR) or by proteomic tools (MALDI-ToF MS). Similar results were observed in other studies, where all *Flavobacterium* isolates recovered from tilapia did not belong to *F. columnare* [10,11,26]. Additionally, to our knowledge, *F. columnare* has not been reported in tilapia according to the new taxonomic classification in the specialized literature. However, this does not imply that this bacterial species is absent in farmed tilapia worldwide or in Brazil, as columnaris disease continues to be reported in various aquaculture fish species in the country [22,42]. On the other hand, it is likely that *F. columnare* is adapted to cause disease in fish species from temperate or cold-water environments, as highlighted in another study [26]. Following the taxonomic reclassification of *Flavobacterium* species, a new diagnostic approach is needed to improve the epidemiological data in Brazil.

Despite this study reporting *Flavobacterium* sp. isolates obtained from six different fish hosts, 47% (8/17) of all clinical cases were related to farmed *O. niloticus*. This may also explain the higher frequency of *F. oreochromis* (35.2%, 6/17) observed in this fish species compared to the others, as well as the high frequency of this pathogen when the observed frequencies are confined to the *O. niloticus* species itself (75%, 6/8). According to LaFrentz et al. [26], *F. oreochromis* would be more frequently associated with columnaris disease in fish species of the Cichlidae family, such as tilapia (*Oreochromis* sp.) and Nile tilapia (*O. niloticus*), farmed in countries in Asia (Thailand, Malaysia, Vietnam, China, and Indonesia) and the Americas (Brazil, Ecuador, Honduras, and Costa Rica). Although *F. oreochromis* was more frequently associated with clinical cases in *O. niloticus*, the observed frequency in diseased *C. macropomum*, a species of fish in the Serrasalmidae family, was also quite significant (29.4%, 5/17). Furthermore, this study presents the first report identifying *F. oreochromis* as causing columnaris disease in native fish, in the species *Brycon amazonicus*, of the Bryconidae family, and in the hybrid *Colossoma macropomum × Piaractus brachypomus*, of the Serrasalmidae family. Considering the higher observed frequency of *F. oreochromis* isolation (76.4%, 13 out of 17) in various diseased fish species from different Brazilian aquaculture locations in this present report and its proven virulence in *O. niloticus*, *C. macropomum*, *Lates calcarifer*, and *Ictalurus punctatus* [12,13,21,43,44], the epidemiological significance of this pathogen in aquaculture fish is noteworthy.

Regarding *F. davisii* and *F. covae*, both bacteria were also isolated from diseased *O. niloticus*, although at low observed frequencies of 11.8 and 5.9%, respectively. The association of columnaris disease with these bacteria has been previously reported [10,11]. However, in this study, these two bacterial species were detected in two different fish from the same clinical case. To our knowledge, this is the first Brazilian report of concurrent infection of *F. davisii* and *F. covae* in a single clinical case of columnaris disease in cultured *O. niloticus*. Similarly, LaFrentz et al. [12] reported the simultaneous detection of *F. davisii*, *F. columnare*, and *F. covae* in diseased channel catfish in aquaculture facilities from the USA, but without isolating these bacteria species. On the other hand, those authors also observed a probable coinfection of *F. columnare* and *F. covae* in an individual diseased *I*. *punctatus* through multiplex PCR. They suggested a possible role for *F. columnare* and *F. davisii* as secondary pathogens following primary infection with *F. covae* in channel catfish. In Nile tilapia, the pathogenicity of *F. oreochromis* and *F. davisii* has already been demonstrated [14,21], but not for *F. covae*. Further studies are needed to elucidate the pathogenic capabilities of *F. covae* and its role in coinfections with other *Flavobacterium* species.

In this study, the only two clinical cases of columnaris disease in siluriformes farmed in Brazil were associated with *F. covae*. In addition, a previous report demonstrated the pathogenic capability of *F. covae* in two species of siluriformes fish from South America [5]. Similarly, reports from the USA have shown a moderate frequency of *F. covae* in diseased *I*. *punctatus*, an important siluriforme species for aquaculture purposes in that country [12,15]. Although this information suggests the epidemiological relevance of *F. covae* in siluriformes, it is important to note that this bacterium has recently been proven pathogenic and associated with outbreaks in perciform fish, such as *L. calcarifer* [45]. Furthermore, experimental challenges with various *F. covae* strains in channel catfish have shown variable pathogenic effects, with mortalities ranging from 0 to 100% [12,15,16]. In contrast, the susceptibility of South American siluriformes of aquaculture importance to *F. davisii* and *F. columnare* has not yet been evaluated, unlike the variable susceptibility observed in channel catfish to these three bacteria [12,21] and the experimental susceptibility of *F. oreochromis* to *Rhamdia quelen* [25], indicating that this area deserves further investigation.

Of the 50 isolates evaluated in this study, only one isolate, PD02, could not be identified as a *Flavobacterium* species using multiplex PCR. However, species identification of this isolate as *F. oreochromis* could be achieved through phylogenetic analysis of the *16S rRNA* gene and MALDI-ToF analysis. The multiplex PCR for *Flavobacterium* species identification is based on the amplification of a region that includes a partial hypothetical protein (targeted by the forward degenerated primer) and an intergenic space (targeted by the reverse degenerated primer) [11]. The failure of multiplex PCR identification could be attributed to a possible lack of DNA integrity. However, this seems unlikely, as the *16S rRNA* gene (~1500 bp) was successfully amplified in two DNA extraction attempts of this isolate, while the expected multiplex PCR amplicon (~659 bp) was not. Another possible explanation for the absence of amplicon generation could be nucleotide variability in the target region for this *F. oreochromis* isolate, which may have prevented primer annealing. Further genomic analysis of this isolate could provide more information about this potential divergent variant.

MALDI-ToF MS has been widely used in the clinical diagnosis of several microorganisms in recent years [46], including emerging etiological agents of infections in fish [36,37,47]. In this study, after the inclusion of MSPs, the *Flavobacterium* sp. isolates were identified with 98.08% accuracy, which was not possible before, since the scores presented were below 1700. The isolate FC32 was the only one that presented a score of 1.913, below the 2.000 required for a reliable species result by MALDI-ToF MS, despite being identified as *F. orechromis* by multiplex PCR. Additionally, other isolates recovered from the same outbreak, clinical case 9, were consistently identified as *F. oreochromis* by both methods. Furthermore, four of these isolates were subsequently analyzed phylogenetically, grouping them within the *F. oreochromis* branch. Although MALDI-ToF MS proved to be a highly accurate tool for identifying the analyzed *Flavobacterium* isolates, species identification was not always possible. This limitation may have arisen from minor variations during growth and sample preparation, which can slightly alter ribosomal protein expression and, consequently, the intensity of m/z peaks used for identification [48]. In our study, the isolates were identified using the standard method (whole bacterial cells), achieving scores close to 2.000. To optimize identification, protein extracts could be used, as they increase the likelihood of detecting specific m/z peaks [49].

In order to verify the results obtained via MALDI-ToF MS and multiplex PCR, all isolates were identified by both methods, with the exception of isolate FC32, identified only by multiplex PCR as *F. oreochromis*, and PD02, identified only by MALDI-ToF MS as *F. oreochromis*, a result that corroborates the species identified by sequencing the *16S rRNA* gene of this isolate, as previously described. The almost perfect agreement is explained by the Kappa value presented in this study, suggesting that both methods can be used to differentiate between *Flavobacterium* species. These data are in line with what was described by LaFrentz et al. [10], where the analyzed strains were identified by MALDI-ToF MS and multiplex PCR. Although LaFrentz et al. achieved a better result using the algorithm model the Supervised Neural Network (SNN) [3], in this study an almost perfect congruence was obtained using the standard Maldi Biotyper software, which even allowed the assignment of isolates to the currently recognized *Flavobacterium* species, allowing the validation of previous reports made with these isolates [7,10,12,36,37]. To make a full validation for species-specific identification of the use of MALDI-ToF MS in *Flavobacterium* in future studies, a more representative number of strains of each species would be necessary.

Overall, the information obtained in this study is valuable for designing control and prevention programs for columnaris-causing bacteria and for encouraging further epidemiologic studies to better understand the impact of columnaris disease in Brazilian aquaculture.

## 5. Conclusions

This study provides evidence of *F. covae*, *F. davisii*, and *F. oreochromis* circulating in various aquaculture facilities across different regions of Brazil by *16S rRNA* gene sequencing, multiplex PCR, and MALDI-ToF MS methods. Additionally, this is the first report of *F. covae* associated with clinical cases in two South American siluriformes species (*Leiarius marmoratus* × *Pseudoplatystoma fasciatum* and *Lophiosilurus alexandri*) and *F. oreochromis* associated with diseased fish from two neotropical families, *Serrasalmidae* (*Colossoma macropomum* × *Piaractus brachypomus*) and *Bryconidae* (*Brycon amazonicus*).

## Figures and Tables

**Table 1:** Identification of strains used in the study; clinical cases; and origin, host, and species results determined by *16S rRNA* gene sequencing, multiplex PCR, and MALDI-ToF MS (before and after inclusion of MSPs).

\	Clinical Case	Origin	Host	Geographical Location	Reference	*16S rRNA* Sequencing			Multiplex PCR	MALDI-ToF MS		
						Species	Identity (%)	Coverage (%)	Species	Before Custom MSP Inclusion †	Organism Best Match	After Custom MSP Inclusion
										Score Value †		Score Value
FC01	1	MG	*O. niloticus*	19°42′23.3″ S + 44°18′46.6″ W	[5]	*F. oreochromis*	98.54 ^a^	100	*F. oreochromis*	1.151	*F. oreochromis*	2.357
FC02	2	MG	*O. niloticus*	21°16′52.4″ S + 45°03′16.7″ W	[5]	*F. oreochromis*	99.64 ^a^	100	*F. oreochromis*	1.218	*F. oreochromis*	2.194
FC03	3	MG	*L. marmoratus × P. fasciatum*	19°54′09.4″ S + 43°59′09.8″ W	[5]	*F. covae*	97.95 ^b^	99	*F. covae*	1.180	*F. covae*	2.212
FC04	3	MG	*L. marmoratus × P. fasciatum*	19°54′09.4″ S + 43°59′09.8″ W	[5]	*F. covae*	99.35 ^c^	99	*F. covae*	1.313	*F. covae*	2.334
FC05	4	MG	*L. alexandri*	19°54′09.4″ S + 43°59′09.8″ W	[5]	*F. covae*	99.50 ^b^	99	*F. covae*	1.324	*F. covae*	2.255
FC06	4	MG	*L. alexandri*	19°54′09.4″ S + 43°59′09.8″ W	[5]	*F. covae*	99.85 ^b^	100	*F. covae*	1.340	*F. covae*	2.157
FC07	5	MG	*O. niloticus*	18°35′26.5″ S + 45°20′10.9″ W	[5]	*F. oreochromis*	99.71 ^a^	100	*F. oreochromis*	1.268	*F. oreochromis*	2.107
FC08	5	MG	*O. niloticus*	18°35′26.5″ S + 45°20′10.9″ W	[5]	*F. oreochromis*	99.43 ^a^	99	*F. oreochromis*	1.332	*F. oreochromis*	2.098
FC09	5	MG	*O. niloticus*	18°35′26.5″ S + 45°20′10.9″ W	[5]	*F. oreochromis*	99.71 ^a^	99	*F. oreochromis*	1.323	*F. oreochromis*	2.022
FC10	5	MG	*O. niloticus*	18°35′26.5″ S + 45°20′10.9″ W	[5]	*F. oreochromis*	98.69 ^a^	100	*F. oreochromis*	1.253	*F. oreochromis*	2.194
FC11	5	MG	*O. niloticus*	18°35′26.5″ S + 45°20′10.9″ W	[5]	*F. oreochromis*	99.78 ^a^	99	*F. oreochromis*	1.222	*F. oreochromis*	2.231
FC12	6	MG	*O. niloticus*	21°18′09.1″ S + 45°53′47.2″ W	[5]	*F. covae*	99.14 ^c^	99	*F. covae*	1.581	*F. covae*	2.214
FC13	6	MG	*O. niloticus*	21°18′09.1″ S + 45°53′47.2″ W	[5]	*F. covae*	99.28 ^c^	99	*F. covae*	1.365	*F. covae*	2.262
FC14	6	MG	*O. niloticus*	21°18′09.1″ S + 45°53′47.2″ W	This study	*F. columnare*	99.21 ^d^	99	*F. davisii*	1.358	*F. davisii*	2.109
FC21	7	RO	*C. macropomum*	8°41′29.1″ S + 63°54′33.2″ W	This study	*F. oreochromis*	99.07 ^a^	99	*F. oreochromis*	1.302	*F. oreochromis*	2.088
FC22	8	MT	*C. macropomum × P. brachypomus*	13°48′59.6″ S + 56°05′32.7″ W	This study	-	-	-	*F. oreochromis*	1.662	*F. oreochromis*	2.087
FC23	8	MT	*C. macropomum × P. brachypomus*	13°48′59.6″ S + 56°05′32.7″ W	This study	*F. oreochromis*	99.85 ^a^	100	*F. oreochromis*	1.196	*F. oreochromis*	2.241
FC24	8	MT	*C. macropomum × P. brachypomus*	13°48′59.6″ S + 56°05′32.7″ W	This study	*F. oreochromis*	100 ^a^	100	*F. oreochromis*	1.307	*F. oreochromis*	2.290
FC25	8	MT	*C. macropomum × P. brachypomus*	13°48′59.6″ S + 56°05′32.7″ W	This study	-	-	-	*F. oreochromis*	1.411	*F. oreochromis*	2.373
FC26	9	RO	*C. macropomum*	8°41′29.1″ S + 63°54′33.2″ W	This study	-	-	-	*F. oreochromis*	1.655	*F. oreochromis*	2.091
FC27	9	RO	*C. macropomum*	8°41′29.1″ S + 63°54′33.2″ W	This study	*F. oreochromis*	98.72 ^e^	99	*F. oreochromis*	1.264	*F. oreochromis*	2.099
FC28	9	RO	*C. macropomum*	8°41′29.1″ S + 63°54′33.2″ W	This study	-	-	-	*F. oreochromis*	1.578	*F. oreochromis*	2.200
FC29	9	RO	*C. macropomum*	8°41′29.1″ S + 63°54′33.2″ W	This study	*F. oreochromis*	98.47 ^a^	100	*F. oreochromis*	1.255	*F. oreochromis*	2.328
FC30	9	RO	*C. macropomum*	8°41′29.1″ S + 63°54′33.2″ W	This study	-	-	-	*F. oreochromis*	1.611	*F. oreochromis*	2.121
FC31	9	RO	*C. macropomum*	8°41′29.1″ S + 63°54′33.2″ W	This study	-	-	-	*F. oreochromis*	1.308	*F. oreochromis*	2.338
FC32	9	RO	*C. macropomum*	8°41′29.1″ S + 63°54′33.2″ W	This study	-	-	-	*F. oreochromis*	1.613	*F. oreochromis*	1.917
FC33	9	RO	*C. macropomum*	8°41′29.1″ S + 63°54′33.2″ W	This study	-	-	-	*F. oreochromis*	1.696	*F. oreochromis*	2.421
FC34	9	RO	*C. macropomum*	8°41′29.1″ S + 63°54′33.2″ W	This study	-	-	-	*F. oreochromis*	1.591	*F. oreochromis*	2.231
FC35	9	RO	*C. macropomum*	8°41′29.1″ S + 63°54′33.2″ W	This study	-	-	-	*F. oreochromis*	1.613	*F. oreochromis*	2.056
FC36	9	RO	*C. macropomum*	8°41′29.1″ S + 63°54′33.2″ W	This study	*F. oreochromis*	99.64 ^a^	99	*F. oreochromis*	1.300	*F. oreochromis*	2.304
FC37	9	RO	*C. macropomum*	8°41′29.1″ S + 63°54′33.2″ W	This study	-	-	-	*F. oreochromis*	1.640	*F. oreochromis*	2.413
FC38	9	RO	*C. macropomum*	8°41′29.1″ S + 63°54′33.2″ W	This study	-	-	-	*F. oreochromis*	1.628	*F. oreochromis*	2.097
FC39	9	RO	*C. macropomum*	8°41′29.1″ S + 63°54′33.2″ W	This study	-	-	-	*F. oreochromis*	1.295	*F. oreochromis*	2.290
FC40	9	RO	*C. macropomum*	8°41′29.1″ S + 63°54′33.2″ W	This study	*F. oreochromis*	99.28 ^a^	99	*F. oreochromis*	1.244	*F. oreochromis*	2.319
FC41	9	RO	*C. macropomum*	8°41′29.1″ S + 63°54′33.2″ W	This study	-	-	-	*F. oreochromis*	1.549	*F. oreochromis*	2.036
FC42	9	RO	*C. macropomum*	8°41′29.1″ S + 63°54′33.2″ W	This study	-	-	-	*F. oreochromis*	1.642	*F. oreochromis*	2.348
FC43	9	RO	*C. macropomum*	8°41′29.1″ S + 63°54′33.2″ W	This study	-	-	-	*F. oreochromis*	1.251	*F. oreochromis*	2.406
FC44	9	RO	*C. macropomum*	8°41′29.1″ S + 63°54′33.2″ W	This study	-	-	-	*F. oreochromis*	1.659	*F. oreochromis*	2.179
FC46	9	RO	*C. macropomum*	8°41′29.1″ S + 63°54′33.2″ W	This study	-	-	-	*F. oreochromis*	1.347	*F. oreochromis*	2.281
FC47	9	RO	*C. macropomum*	8°41′29.1″ S + 63°54′33.2″ W	This study	-	-	-	*F. oreochromis*	1.525	*F. oreochromis*	2.026
FC48	9	RO	*C. macropomum*	8°41′29.1″ S + 63°54′33.2″ W	This study	-	-	-	*F. oreochromis*	1.608	*F. oreochromis*	2.220
FC49	10	MG	*O. niloticus*	19°54′09.4″ S + 43°59′09.8″ W	This study	-	-	-	*F. davisii*	1.580	*F. davisii*	2.082
FC50	10	MG	*O. niloticus*	19°54′09.4″ S + 43°59′09.8″ W	This study	*F. columnare*	98.91 ^d^	100	*F. davisii*	1.348	*F. davisii*	2.252
PD01	11	SP	*O. niloticus*	23°39′8.212″ S + 47°13′17.9″ W	This study	*F. oreochromis*	99.85 ^a^	99	*F. oreochromis*	1.312	*F. oreochromis*	2.361
PD02	12	SP	*O. niloticus*	23°39′8.212″ S + 47°13′17.9″ W	This study	*F. oreochromis*	99.49 ^a^	99	NA*	1.255	*F. oreochromis*	2.328
PD03	13	SP	*O. niloticus*	23°39′8.212″ S + 47°13′17.9″ W	This study	*F. oreochromis*	99.78 ^a^	100	*F. oreochromis*	1.282	*F. oreochromis*	2.325
AM-FC08	14	AM	*C. macropomum*	2°30′32.695″ S + 59°36′1.8″ W	[25]	*F. oreochromis*	98.97 ^a^	99	*F. oreochromis*	1.192	*F. oreochromis*	2.354
AMFC30	15	AM	*C. macropomum*	1°13′14.862″ S + 60°1′22.8″ W	[17]	*F. oreochromis*	99.85 ^a^	99	*F. oreochromis*	1.232	*F. oreochromis*	2.196
AM-FC42	16	SP	*C. macropomum*	20°47′1.05″ S + 49°21′34.2″ W	This study	*F. oreochromis*	99.42 ^a^	99	*F. oreochromis*	1.548	*F. oreochromis*	2.060
AM-FC56	17	AM	*B. amazonicus*	3°6′26.669″ S + 59°58′36.545″ W	This study	*F. oreochromis*	99.93 ^a^	99	*F. oreochromis*	1.373	*F. oreochromis*	2.113
ATCC23463	-	USA	*Oncorynchus tshawytscha*		[28]	*F. columnare*	99.93 ^f^	100	*F. columnare*	1.441	*F. columnare*	2.057
ATCC49512	-	France	*Salmo truta*		[29]	*F. columnare*	99.57 ^g^	100	*F. columnare*	1.562	*F. columnare*	2.601

Lowercase letters indicate strains used for NCBI identities and their accession numbers: ^a^ TI2056 (KX711900.1); ^b^ AL-02-36 (MW353013.1); ^c^ C#2 (CP015107.1); ^d^ CUVET1215 (KF274040.1); ^e^ TI1690 (KX711899.1); ^f^ B185R (CP013012.1); ^g^ 2023717G (OR946449.1). AM, Amazonas State; MG, Minas Gerais State; MT, Mato Grosso State; SP, São Paulo State; RO, Rondônia State. NA*, No amplicon detected. †, Indicates that the scores were less than 1.700 and that the strains were considered as “not reliably identified”.

**Table 2:** Frequency of identified *Flavobacterium* species by origin and host. Percentage values are relative to the total clinical cases (N = 17).

		*Flavobacterium Species* (Number of Clinical Cases; Percentage)				
		*F. oreochromis*	*F. covae*	*F. davisii*	Concurrent Infection (*F. covae* and *F. davisii*)	Total
Origin	AM	*n* = 3; 17.6%	-	-	-	*n* = 3; 17.6%
	MG	*n* = 3; 17.6%	*n* = 2; 11.8%	*n* = 1; 5.9%	*n* = 1; 5.9%	*n* = 7; 41.2%
	MT	*n* = 1; 5.9%	-	-	-	*n* = 1; 5.9%
	RO	*n* = 2; 11.8%	-	-	-	*n* = 2; 11.8%
	SP	*n* = 4; 23.5%	*-*	-	-	*n* = 4; 23.5%
Host	*O. niloticus*	*n* = 6; 35.2%	*-*	*n* = 1; 5.9%	*n* = 1; 5.9%	*n* = 8; 47.0%
	*C. macropomum*	*n* = 5; 29.4%	-	-	-	*n* = 5; 29.4%
	*B. amazonicus*	*n* = 1; 5.9%	-	-	-	*n* = 1; 5.9%
	*L. alexandri*	-	*n* = 1; 5.9%	-	-	*n* = 1; 5.9%
	*C. macropomum × P. brachypomus*	*n* = 1; 5.9%	-	-	-	*n* = 1; 5.9%
	*L. marmoratus × P. fasciatum*	-	*n* = 1; 5.9%	-	-	*n* = 1; 5.9%
	Total	*n* = 13; 76.4%	*n* = 2; 11.8%	*n* = 1; 5.9%	*n* = 1; 5.9%	*n* = 17; 100%

AM, Amazonas State; MG, Minas Gerais State; MT, Mato Grosso State; SP, São Paulo State; RO, Rondônia State.

## Data Availability

The sequences used in this study are available on NCBI under the following accession numbers: *F. oreochromis* (PQ578965, PQ578966, PQ578968, PQ578969, PQ578970, PQ578971, PQ578973, PQ578974, PQ578975, PQ578976, PQ578977, PQ578978, PQ578983, PQ578984, PQ578985, PQ578986, PQ578987, PQ578988, PQ578989, PQ578990, PQ578991, PQ578992, and PQ578993), *F. davisii* (PQ578967 and PQ578972), and *F. covae* (PQ578979, PQ578980, PQ578981, and PQ578982).

## Supplementary Materials

The following supporting information can be downloaded at https://www.mdpi.com/article/10.3390/pathogens14030219/s1: Table S1: BLAST identities of Brazilian *F. columnare* isolates based on sequencing of multiplex amplicons. Table S2: Sequences of primers used for the multiplex PCR for Flavobacterium species, including amplicon sizes.
