# Supplementary material for: Identification and Phylogenetic Analysis of Flavobacterium spp. Associated with Aquaculture Fish Diseased from Brazil
Source: Pathogens. 2025 Feb 22;14(3):219. doi: 10.3390/pathogens14030219 (PMC11944601; doi:10.3390/pathogens14030219)
Supplement: Supplementary file 1 [file pathogens-14-00219-s001.zip › pathogens-3463045-supplementary.pdf]

# Supplementary

**Supplementary Table S1:** BLAST analysis of Brazilian isolates, previously identified as *F. columnare*, based on sequencing of multiplex amplicon**.**

| **Isolate** (Accession Number) | **Amplicon size (bp)** | **Identity (%)***(Species, Id NCBI strain, Accession Number) | **Coverage (%)** |
|---|---|---|---|
| FC14 (PQ621430) | 913 | 97.31 (*F. davisii*, strain 90-106, CP067378.1) | 97 |
| FC50 (PQ621432) | 900 | 98.07 (*F. davisii*, strain 90-106, CP067378.1) | 98 |
| FC07 (PQ621429) | 680 | 98.52 (*F. oreochromis*, strain Costa Rica 04-02-TN, CP067377.1) | 98 |
| FC27 (PQ621431) | 672 | 99.95 (*F. oreochromis*, strain Costa Rica 04-02-TN, CP067377.1) | 99 |
| FC04 (PQ621427) | 328 | 99.06 (*F. covae*, strain C#2, CP015107.1) | 97 |
| FC05 (PQ621428) | 338 | 96.64 (*F. covae*, strain C#2, CP015107.1) | 96 |

*Identity based on the lowest E-value displayed after BLAST procedure (updated at 26/06/2024).

**Supplementary Table S2:** Sequences of primers used for the multiplex PCR to Flavobacterium species, including amplicon sizes. Information adapted from La Frentz et al., 2022 and LaFrentz et al., 2019 [10,11].

| Primer | Targeted Flavobacterium species | Amplicon size (bp) | Sequence (5′ → 3′) |
|---|---|---|---|
| GG-fwd | All | – | ACRGGRGATAAAGCAGAASA |
| GG1-rev | *F. columnare* | 415 | GACTTTTGTGTTGAAACGG |
| GG2-rev | *F. covae* | 320 | AAGAAAATAGGGGAGAGG |
| GG3-rev | *F. davisii* | 894 | CAAGTTTCGTTATGATGAGG |
| GG4-rev | *F. oreochromis* | 659 | TCCAAAAAGTCCGMAATC |
